# Exercise-induced hypertension, atherogenic lipid subfractions, and cardiorespiratory fitness in marathon runners

**DOI:** 10.1016/j.pmedr.2025.103326

**Published:** 2025-11-29

**Authors:** Eun Sun Yoon, Jong-Young Lee, Young-Joo Kim

**Affiliations:** a Department of Sports for All, Korea National Open University, Seoul 02844, Republic of Korea; b Division of Cardiology, Department of Internal Medicine, Cardiovascular Center, Hallym University College of Medicine, Hallym University Sacred Heart Hospital, Gyeonggi-do 14068, Republic of Korea; c School of Sports Science, Sungshin Women's University, Seoul 02844, Republic of Korea

**Keywords:** Exercise-induced hypertension, Small dense low-density lipoprotein, Cardiorespiratory fitness, Lipid subfractions, Endurance athletes, Cardiovascular risk

## Abstract

**Objective:**

Exercise-induced hypertension (EIH), defined by an exaggerated exercise systolic blood pressure response, has been linked to cardiovascular events. This study examined whether EIH is associated with atherogenic lipid subfractions and whether cardiorespiratory fitness (CRF) relates to lipid particle characteristics in male marathon runners.

**Methods:**

In this cross-sectional study, 51 male marathon runners aged 40–65 years were tested between March and September 2023 at the Exercise Physiology Laboratory, Sungshin Women's University, Seoul, South Korea. Participants were classified as having EIH (*n* = 29, maximal systolic blood pressure ≥ 210 mmHg) or a normal response (*n* = 22). Lipid subfractions, including small dense low-density lipoprotein (LDL), were assessed by polyacrylamide gel electrophoresis, and CRF was measured by maximal oxygen uptake (VO₂max).

**Results:**

Lipid subfractions did not differ between EIH and normotensive runners, including small dense LDL and mean LDL particle size (all *p* > 0.05). Higher VO₂max was associated with lower small dense LDL (*r* = −0.41, *p* < 0.01) and triglycerides (*r* = −0.36, p < 0.01), and higher HDL-cholesterol (*r* = 0.33, *p* = 0.02) and mean LDL particle size (*r* = 0.39, p < 0.01).

**Conclusions:**

Among male marathon runners, EIH was not associated with adverse atherogenic lipid subfractions, whereas higher CRF correlated with more favorable lipid particle profiles, suggesting fitness-related metabolic adaptations may mitigate lipid-mediated cardiovascular risk.

## Highlights

• Exercise-induced hypertension was not linked to harmful lipid subfractions.
• Higher fitness levels were associated with more favorable blood lipid patterns.
• Runners with higher fitness showed much lower atherogenic lipid phenotype risk.
• Exercise-induced hypertension may increase organ risk through non-lipid pathways.

## 1 Introduction

Exercise-induced hypertension (EIH), defined by an exaggerated systolic blood pressure (SBP) response during progressive exercise testing (typically ≥210 mmHg in men), represents a significant cardiovascular phenotype with emerging clinical relevance (Xinwen et al., 2024; Zografos et al., 2025). This hemodynamic abnormality occurs despite normal resting blood pressure values and has been identified as a predictor of future cardiovascular complications (Kim and Ha, 2016; Miyai et al., 2000). Recent evidence suggests that EIH may increase the prevalence of coronary artery plaque among middle-aged male marathon runners, indicating its potential role as a cardiovascular risk factor even in highly trained populations (Kim et al., 2020).

The pathophysiological mechanisms underlying EIH encompass enhanced sympathetic nervous system activation, increased cardiac output with inadequate peripheral vasodilation, elevated arterial stiffness, and endothelial dysfunction (Kim and Park, 2024; Schultz et al., 2013). Contemporary cardiovascular risk assessment increasingly recognizes the importance of advanced lipid phenotyping beyond traditional cholesterol measurements. Low-density lipoprotein (LDL) particles demonstrate significant heterogeneity in size, density, and atherogenic potential (Hoogeveen et al., 2014; Qi et al., 2020). Small dense LDL (sdLDL) particles, characterized by their enhanced arterial wall penetration, increased oxidative susceptibility, and prolonged plasma residence time, demonstrate markedly greater atherogenicity compared to larger, buoyant LDL particles (Santos et al., 2020; Jin et al., 2020; Imamura et al., 2023). The metabolic pathway involves hepatic lipase activity and cholesteryl ester transfer protein-mediated lipid exchange generating smaller, denser particles from triglyceride-enriched precursors (Rizvi et al., 2014). These particles exhibit impaired LDL receptor binding, resulting in prolonged exposure to oxidative modification and preferential retention in the arterial intima, initiating atherosclerotic plaque development (Rizvi et al., 2021). Furthermore, sdLDL induces endothelial dysfunction, promotes inflammatory responses, and enhances platelet aggregation (Nibali et al., 2015).

Prospective epidemiological studies have established sdLDL as an independent predictor of coronary heart disease, with predictive value that may exceed conventional LDL-cholesterol measurements (Hoogeveen et al., 2014).

Cardiorespiratory fitness (CRF), quantified through maximal oxygen uptake (VO₂max), represents one of the most potent predictors of cardiovascular outcomes (Ross et al., 2016; Kaminsky et al., 2022). High CRF exerts profound favorable effects on lipoprotein metabolism through multiple mechanisms including enhanced lipoprotein lipase activity, improved Very low-density lipoprotein (VLDL) clearance, and increased LDL particle size (Wang and Xu, 2017; Halverstadt et al., 2007). A landmark randomized controlled trial demonstrated that improved CRF correlates with changes in the number and size of small dense LDL particles, with exercise training and dietary instruction leading to qualitative and quantitative improvements in LDL characteristics (Kawano et al., 2009). Meta-analyses consistently demonstrate that regular aerobic exercise training promotes a shift toward larger, less atherogenic LDL particles while simultaneously reducing sdLDL concentrations (Sarzynski et al., 2015; Kraus et al., 2002). However, the potential for high CRF to modulate the relationship between exercise-induced hemodynamic abnormalities and atherogenic lipid profiles remains unexplored.

Marathon runners represent a unique population exhibiting the coexistence of exceptional CRF (cardiovascular protective factor) and relatively high EIH prevalence (potential cardiovascular risk factor). Interestingly, studies have shown that marathon participation is associated with lower prevalence of hypertension, hypercholesterolemia, and diabetes, with these benefits increasing with frequency of marathon participation (Leyk et al., 2009). This paradoxical combination provides an ideal model for investigating whether fitness-mediated metabolic adaptations can attenuate lipid-related cardiovascular risks independent of exercise-induced blood pressure responses. However, the relationships among EIH, atherogenic lipid subfractions, and cardiorespiratory fitness are not well understood.

Therefore, this investigation aimed to: (1) compare atherogenic lipid subfraction profiles between marathon runners with and without EIH; (2) examine associations between CRF and comprehensive lipid particle characteristics; and (3) determine whether CRF modulates relationships between exercise blood pressure responses and atherogenic lipid profiles. We hypothesized that: (1) marathon runners with EIH would demonstrate more atherogenic lipid profiles, characterized by elevated sdLDL and intermediate-density lipoproteins (IDL)-B, IDL-C concentrations, compared to normotensive counterparts; and (2) superior CRF would associate with favorable lipid particle characteristics, potentially attenuating EIH-related lipid abnormalities.

## 2 Materials and methods

### 2.1 Study design

This cross-sectional study was conducted between March and September 2023 at the Exercise Physiology Laboratory, Sungshin Women's University, Seoul, South Korea. The study examined associations between exercise-induced hypertension, atherogenic lipid subfractions, and cardiorespiratory fitness in recreational male marathon runners. The study protocol received institutional review board approval (SSWUIRB 20230921-091) and all procedures adhered to the principles of the Declaration of Helsinki. All participants provided written informed consent prior to participation.

### 2.2 Participant characteristics

Recreational male marathon runners aged 40–65 years were recruited from local running clubs and marathon events through flyers and online announcements. Eligibility criteria included a history of completing at least one full marathon in the past two years, absence of physician-diagnosed cardiovascular or metabolic disease, and no use of antihypertensive or lipid-lowering medications. Men with musculoskeletal conditions limiting exercise testing or with incomplete laboratory measurements were excluded.

A total of 51 eligible runners were enrolled and completed all assessments. Based on peak exercise systolic blood pressure during treadmill testing, 29 participants (56.9 %) were classified as having exercise-induced hypertension and 22 (43.1 %) as having a normal blood pressure response. Baseline characteristics, including age, anthropometric measures, training history, and cardiorespiratory fitness, are presented in Table 1. The exercise-induced hypertension group showed higher resting systolic blood pressure and resting heart rate, as well as markedly elevated exercise blood pressure responses compared with the normotensive group.

**Table 1:** Characteristics of male marathon runners aged 40–65 years in 2023, Exercise Physiology Laboratory, Sungshin Women's University, Seoul, South Korea.

Variables	EIHG (n = 29)	NTG (n = 22)	*p*-value
Age (yr)	56.5 ± 6.8	58.0 ± 6.5	0.41
Height (cm)	171.2 ± 7.7	170.6 ± 7.0	0.77
Weight (kg)	66.8 ± 8.3	66.4 ± 7.2	0.93
BMI (kg/m^2^)	22.8 ± 2.5	22.8 ± 1.9	0.99
RSBP (mmHg)	125.9 ± 12.3	119.1 ± 8.9	0.05
RDBP (mmHg)	77.9 ± 7.0	76.1 ± 5.8	0.46
RHR (bpm)	60.2 ± 7.7	55.4 ± 6.9	0.03
MSBP (mmHg)	224.3 ± 10.6	192.0 ± 6.8	<0.01
MDBP (mmHg)	94.4 ± 9.4	86.4 ± 6.1	<0.01
MHR (bpm)	168.9 ± 9.7	161.7 ± 14.5	0.02
VO_2_max (ml/kg/min)	48.3 ± 6.7	48.4 ± 7.2	0.67
GXT time (sec)	811.7 ± 74.8	799.1 ± 120.7	0.65
Exercise Characteristics			
Marathon career (yr)	16.6 ± 6.8	19.3 ± 8.0	0.07
Marathon completed (number)	46.2 ± 43.6	80.4 ± 59.4	0.04
Marathon completed record (min)	228.1 ± 37.0	224.0 ± 34.2	0.71

Data are presented as mean ± standard deviation for normally distributed variables, median (interquartile range) for non-normally distributed variables, and number (percentage) for categorical variables. *P*-values are from independent samples t-tests for normally distributed continuous variables, Mann–Whitney U tests for non-normally distributed continuous variables, and chi-square tests for categorical variables. EIHG: Exercise-induced hypertension group, NTG; Normotensive group, BMI; body mass index, RSBP; resting systolic blood pressure, RDBP; resting diastolic blood pressure, RHR; resting heart rate, BPM; beat per minute, MSBP; maximal systolic blood pressure, MDBP; maximal diastolic blood pressure, MHR; maximal heart rate, GXT; grade exercise test.

### 2.3 Experimental procedures

Pre-testing Protocol: Participants abstained from vigorous exercise for 48 h, caffeine for 12 h, and food/beverages (except water) for 12 h before testing. All assessments were conducted between 7:00 and 10:00 AM to minimize circadian variations.

### 2.4 Measures

#### 2.4.1 Anthropometric and resting blood pressure measures

Height and body mass were measured using calibrated equipment. Resting blood pressure was assessed following standardized guidelines using validated automated sphygmomanometry (WatchBP Home™ 3MX1-1, Microlife AG, Switzerland). After a 10-min quiet rest in a seated position, three measurements were obtained at 2-min intervals, with the average of the final two measurements recorded.

#### 2.4.2 Maximal exercise testing and blood pressure measures

Symptom-limited graded exercise testing was performed using the Bruce protocol on a motorized treadmill (Q-Stress®, Quinton Cardiology Systems, Bothell, WA, USA). Continuous 12‑lead electrocardiography monitoring was maintained throughout testing. VO₂max was measured using a calibrated metabolic cart (TrueOne® 2400, ParvoMedics, Sandy, UT, USA) with breath-by-breath gas analysis. Data were averaged over 15-s intervals with VO₂max determined as the highest 30-s averaged value meeting established criteria. Automated blood pressure assessment (Tango®M, SunTech Medical, Morrisville, NC) was performed during the final 30 s of each 3-min stage. A trained researcher used an electronic stethoscope to minimize measurement errors and ensure accurate identification of Korotkoff sounds. Peak exercise SBP was defined as the highest value achieved during maximal testing. Participants were categorized as EIH-positive (peak SBP ≥ 210 mmHg) or normotensive (peak SBP < 210 mmHg) based on established clinical criteria (Lauer et al., 1992). The criteria for termination and maximal effort of exercise tests followed the guidelines of the American College of Sports Medicine (2009) (respiratory exchange ratio of ≥1.10, a plateau in oxygen uptake (VO_2_), achievement of 90–100 % of age-predicted maximal heart rate, or a rating of perceived exertion of 17 or higher on the Borg scale are met.

#### 2.4.3 Lipid subfraction profiles analysis

Fasting venous blood samples (20 mL) were obtained from the antecubital vein after 8–12 h of fasting (Naugler and Sidhu, 2014). Samples were collected in EDTA-containing tubes, immediately placed on ice, and centrifuged at 3000 rpm for 15 min at 4 °C. Plasma was separated and stored at −80 °C until analysis. Conventional lipids (total cholesterol [TC], triglyceride [TG], HDL-cholesterol [HDL-C], and LDL-cholesterol [LDL-C]) were measured using enzymatic colorimetric assays on an automated analyzer (GC labs, Yongin, Korea). LDL-C was calculated using the Friedewald equation when TG < 400 mg/dL.

Lipoprotein subfraction analysis was performed using the Quantimetrix Lipoprint™ System (Quantimetrix Corporation, Redondo Beach, CA, USA), employing 3 % polyacrylamide gel electrophoresis for size-based particle separation. Separated lipoproteins were classified into distinct subfractions. Electrophoresis was conducted at constant voltage, followed by densitometric scanning at 610 nm using the Helena EDC system. Lipoproteins were classified into VLDL, IDL-A, IDL-B, IDL-C, LDL subfractions 1–5. Mean LDL particle size was calculated based on weighted subfraction distribution. LDL subfraction score was calculated by multiplying each subfraction's concentration by its assigned weight and dividing by total LDL cholesterol, with higher values indicating greater sdLDL predominance. Atherogenic phenotype was defined as mean LDL particle size <26.8 nm (atherogenic phenotype) or ≥26.8 nm (non-atherogenic phenotype). Large LDL comprised subfractions 1–2; small dense LDL (sdLDL) comprised subfractions 3–5.

### 2.5 Statistical analysis

Data normality was assessed using Shapiro-Wilk tests. Independent samples *t*-tests were used for normally distributed continuous variables, while Mann-Whitney *U* tests were employed for non-normal distributions. Chi-square tests assessed categorical variable differences. Pearson correlation coefficients were calculated for normally distributed variables, while Spearman's rank correlation was used for non-normal distributions. Group differences in lipid profiles and atherogenic subfractions were examined using univariate general linear models (analysis of covariance), with group (exercise-induced hypertension vs normotensive, or high- vs low-fitness) as the fixed factor and the number of completed marathons as a covariate. All analyses were conducted using SPSS version 29.0 (IBM Corporation, Armonk, NY, USA) with significance set at *p* < 0.05.

## 3 Results

### 3.1 Lipid profiles and atherogenic subfractions by EIH status

Comprehensive lipid analysis revealed no significant differences in conventional lipid parameters between EIH and normotensive groups, including total cholesterol, triglycerides, HDL-cholesterol, and LDL-cholesterol (all *p* > 0.05) (Table 2). Despite marked differences in exercise blood pressure responses, atherogenic lipid subfraction profiles were remarkably similar between groups. No significant differences were observed in sdLDL concentrations, IDL-B levels (8.7 ± 3.6 vs. 10.1 ± 2.7 mg/dL, *p* = 0.09) or mean LDL particle size (268.7 ± 3.4 vs. 268.0 ± 3.7 nm, *p* = 0.48). The weighted LDL subfraction score was also comparable between groups (1.6 ± 0.3 vs. 1.7 ± 0.4, *p* = 0.75).There was no statistically significant difference in the prevalence of atherogenic phenotype between the EIHG and NTG (*p* = 0.80).

**Table 2:** Comparison of lipid profiles and atherogenic subfractions by exercise-induced hypertension status, controlling for completed marathons, in male marathon runners aged 40–65 years, Exercise Physiology Laboratory, Sungshin Women's University, Seoul, South Korea, 2023.

Variables	EIHG (*n* = 29)	NTG (*n* = 22)	p-value
Conventional lipids			
TC (mg/dL)	190.4 ± 42.3	200.1 ± 46.6	0.94
TG (mg/dL)	82 0.0 [61.5–117.5]	86.0 [64.0–113.3]	0.76
HDL-C (mg/dL)	59.9 ± 15.1	60.5 ± 18.2	0.88
LDL-C (mg/dL)	111.5 ± 32.9	120.5 ± 33.7	0.29
Atherogenic subfractions			
VLDL (mg/dL)	21.8 ± 7.2	21.1 ± 9.2	0.78
IDLA (mg/dL)	10.0 [9.0–14.0]	11.0 [9.0–14.3]	0.46
IDLB (mg/dL)	8.7 ± 3.6	10.1 ± 2.7	0.09
IDLC (mg/dL)	23.4 ± 7.2	27.2 ± 6.9	0.05
LDL subfractions			
Large LDL (LDL 1–2, mg/dL)	59.5 ± 16.0	63.5 ± 20.9	0.39
Small dense LDL (LDL 3–5, mg/dL)	3.0 [2.0–7.0]	4.0 [2.0–8.8]	0.81
Mean LDL particle size (nm)	270.0 [267.0–271.0]	269.0 [266.8–270.0]	0.48
LDL subfraction score	1.5 [1.4–1.8]	1.6 [1.5–1.7]	0.75
Atherogenic phenotype (n, %)	8 (27.6 %)	8 (36.4 %)	0.82

Data are presented as mean ± standard deviation for normally distributed variables, median (interquartile range) for non-normally distributed variables, and number (percentage) for categorical variables. *P*-values are from univariate general linear models (analysis of covariance) comparing exercise-induced hypertension and normotensive groups, with the number of completed marathons included as a covariate. EIHG; Exercise-induced hypertension group, NTG; Normotensive group, TC; total cholesterol, TG; triglyceride, HDL-C; high density lipoprotein cholesterol, LDL-C; low density lipoprotein cholesterol, VLDL; very low density lipoprotein, IDL: intermediate density lipoprotein.

### 3.2 Cardiorespiratory fitness and lipid particle characteristics

Participants were stratified by median VO₂max (48.2 mL/kg/min) into high-fitness (HFG, *n* = 26) and low-fitness (LFG, *n* = 25) groups. The HFG exhibited significantly superior cardiorespiratory fitness (53.3 ± 4.3 vs. 43.1 ± 1.8 mL/kg/min, *p* < 0.01) and lower adiposity (BMI: 21.7 ± 1.5 vs. 24.0 ± 2.3 kg/m^2^, p < 0.01), with no differences in age or resting hemodynamics (Table 3). Conventional lipids were comparable between groups (all *p* > 0.05). However, atherogenic lipoprotein subfractions revealed marked differences. The HFG displayed larger mean LDL particle size (median: 270.0 [269.0–271.0] vs. 268.0 [264.0–270.5] nm, *p* = 0.03), lower small dense LDL (sdLDL) concentration (3.0 [2.0–4.0] vs. 4.0 [2.5–15.0] mg/dL, *p* = 0.01), and reduced LDL subfraction score (1.5 [1.4–1.6] vs. 1.6 [1.5–2.0], *p* = 0.04). Atherogenic phenotype prevalence was threefold lower in the HFG (15.4 % vs. 48.0 %, p = 0.01), highlighting a strong protective association between high cardiorespiratory fitness and favorable LDL particle characteristics, independent of lifetime marathon completions.

**Table 3:** Comparison of lipid profiles and atherogenic subfractions by fitness levels, controlling for completed marathons, in male marathon runners aged 40–65 years, Exercise Physiology Laboratory, Sungshin Women's University, Seoul, South Korea, 2023.

Variable	HFG (N = 26)	LFG (*N* = 25)	p-value
VO_2_max (mL/kg/min)	53.3 ± 4.3	43.1 ± 1.8	<0.01
Age (year)	59.5 [54.0–62.3]	59.0 [52.0–62.5]	0.93
Height (cm)	170.3 ± 6.2	171.6 ± 8.4	0.54
Weight (kg)	62.9 ± 5.5	70.5 ± 8.0	<0.01
BMI (kg/m^2^)	21.7 ± 1.5	24.0 ± 2.3	<0.01
RSBP (mmHg)	120.0 [110.0–130.0]	125.0 [115.0–135.0]	0.06
RDBP (mmHg)	80.0 [70.0–80.0]	80.0 [70.0–85.0]	0.49
RHR (bpm)	57.2 ± 7.2	59.1 ± 8.2	0.37
MSBP (mmHg)	210.0 ± 17.8	210.7 ± 19.7	0.90
MDBP (mmHg)	91.3 ± 9.3	90.7 ± 8.9	0.77
MHR (bpm)	166.2 ± 10.7	165.4 ± 14.2	0.85
Conventional lipids			
TC (mg/dL)	190.4 ± 42.3	199.0 ± 46.2	0.49
TG (mg/dL)	86.6 ± 38.9	117.8 ± 81.1	0.15
HDLC (mg/dL)	61.6 ± 16.6	58.6 ± 16.3	0.52
LDLC (mg/dL)	111.5 ± 31.2	119.5 ± 35.3	0.40
Atherogenic subfractions			
VLDL (mg/dL)	20.3 ± 7.2	22.7 ± 8.8	0.30
IDLA (mg/dL)	12.0 [9.2–14.0]	10.0 [8.5–12.0]	0.61
IDLB (mg/dL)	8.6 ± 3.0	10.1 ± 3.3	0.11
IDLC (mg/dL)	24.3 ± 6.6	25.9 ± 7.9	0.45
LDL subfractions			
Large LDL (LDL 1–2, mg/dL)	60.2 ± 17.5	62.3 ± 19.2	0.70
Small dense LDL (LDL 3–5, mg/dL)	3.0 [2.0–4.0]	4.0 [2.5–15.0]	0.01
Mean LDL particle size (nm)	270.0 [269.0–271.0]	268.0 [264.0–270.5]	0.03
LDL subfraction score	1.5 [1.4–1.6]	1.6 [1.5–2.0]	0.04
Atherogenic phenotype (n, %)	4 (15.4 %)	12 (48.0 %)	0.01

Data are presented as mean ± standard deviation for normally distributed variables, median (interquartile range) for non-normally distributed variables, and number (percentage) for categorical variables. *P*-values are from univariate general linear models (analysis of covariance) comparing high- and low-fitness groups, with the number of completed marathons included as a covariate. HFG; high fitness group, LFG; low fitness group, BMI; body mass index, RSBP; resting systolic blood pressure, RDBP; resting diastolic blood pressure, RHR; resting heart rate, BPM; beat per minute, MSBP; maximal systolic blood pressure, MDBP; maximal diastolic blood pressure, MHR; maximal heart rate, TC; total cholesterol, TG; triglyceride, HDL-C; high density lipoprotein cholesterol, LDL-C; low density lipoprotein cholesterol, VLDL; very low density lipoprotein, IDL: intermediate density lipoprotein.

### 3.3 Correlation analysis: blood pressure, fitness, and lipid relationships

**Fig. 1:**
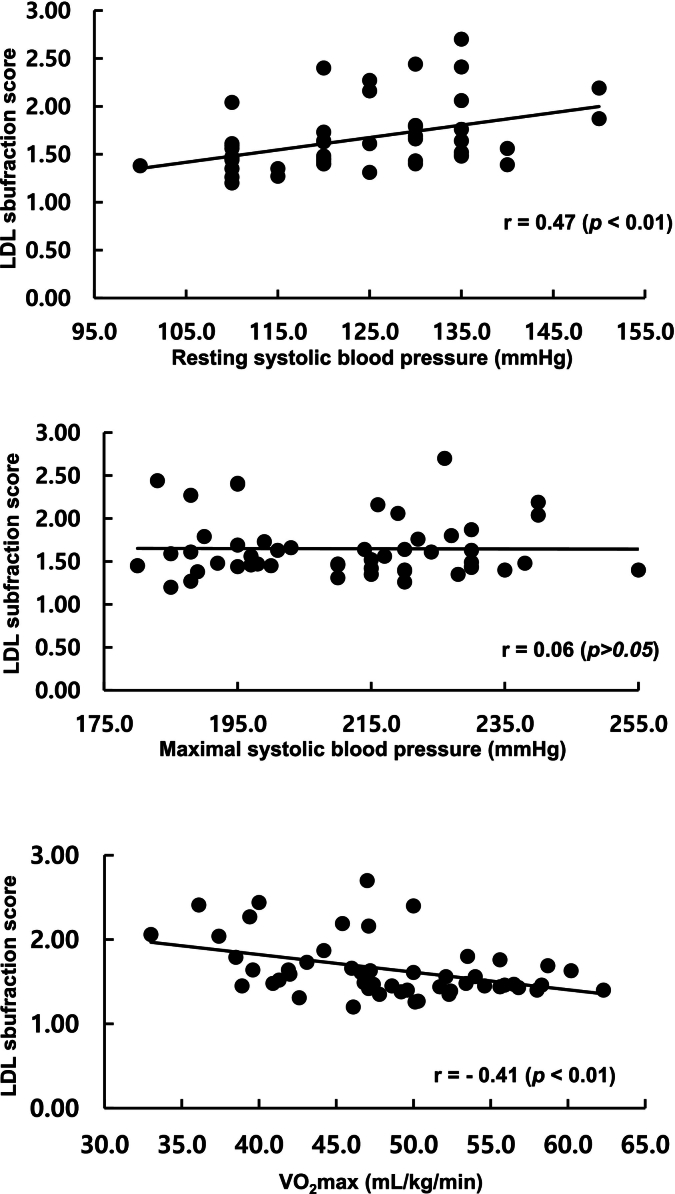
Correlation between resting systolic blood pressure, maximal systolic blood pressure, maximal oxygen uptake, and low-density lipoprotein subfraction score in male marathon runners aged 40–65 years, Exercise Physiology Laboratory, Sungshin Women's University, Seoul, South Korea, 2023.

Comprehensive correlation analysis revealed distinct patterns of association between hemodynamic parameters, fitness, and lipid characteristics (Table 4 and Fig. 1). Maximal SBP(MSBP) showed no significant correlations with any atherogenic lipid parameter (all *p* > 0.05), supporting the absence of association between EIH and adverse lipid profiles. Conversely, resting SBP(RSBP) was positively correlated with TG (*r* = 0.43, *p* < 0.01), VLDL (*r* = 0.38, *p* = 0.01), LDL2 (*r* = 0.34, *p* = 0.02), LDL3 (r = 0.43, p < 0.01), LDL4 (*r* = 0.37, p = 0.01), and LDL subfraction score (*r* = 0.47, *p* ≤ 0.01), and inversely correlated with HDL-cholesterol (*r* = −0.35, p = 0.01) and mean LDL particle size (*r* = −0.46, p < 0.01). Cardiorespiratory fitness (VO₂max) was positively correlated with HDL-cholesterol (*r* = 0.33, p = 0.02) and mean LDL particle size (*r* = 0.39, *p* ≤0.01), and inversely correlated with TG (*r* = −0.36, p = 0.01), VLDL (*r* = −0.28, *p* = 0.05), LDL subfraction score (*r* = −0.41, p < 0.01).

**Table 4:** Correlations between systolic blood pressure, cardiorespiratory fitness, and lipid profiles in male marathon runners aged 40–65 years, Exercise Physiology Laboratory, Sungshin Women's University, Seoul, South Korea, 2023.

Variable	RSBP	MSBP	VO_2_max
RSBP (mmHg)	–	0.387^⁎⁎^	−0.28^⁎^
MSBP (mmHg)		–	0.05
TC (mg/dL)	0.21	0.01	−0.03
TG (mg/dL)	0.43^⁎⁎⁎^	0.07	−0.36^⁎⁎^
HDLC (mg/dL)	−0.35^⁎⁎^	0.08	0.33^⁎⁎^
LDLC (mg/dL)	0.16	−0.06	−0.08
VLDL (mg/dL)	0.38^⁎⁎^	0.12	−0.28^⁎^
IDLA (mg/dL)	0.09	−0.04	0.01
IDLB (mg/dL)	0.27^⁎^	−0.17	−0.26
IDLC (mg/dL)	0.26	−0.17	−0.17
LDL1 (mg/dL)	−0.17	−0.00	0.21
LDL2 (mg/dL)	0.34^⁎^	−0.04	−0.15
LDL3 (mg/dL)	0.43^⁎⁎^	0.02	−0.36^⁎⁎^
LDL4 (mg/dL))	0.37^⁎⁎^	0.06	−0.25
Mean LDL particle size (nm)	−0.46^⁎⁎⁎^	−0.13	0.39^⁎⁎^
LDL subfraction score	0.47^⁎⁎⁎^	0.06	−0.41^⁎⁎^

Correlation coefficients are Pearson's r for normally distributed variables and Spearman's rank correlation coefficients for non-normally distributed variables. *P*-values correspond to the respective correlation tests. RSBP; resting systolic blood pressure, MSBP; maximal systolic blood pressure, TC; total cholesterol, TG; triglyceride, HDL-C; high density lipoprotein cholesterol, LDL-C; low density lipoprotein cholesterol, VLDL; very low density lipoprotein, IDL: intermediate density lipoprotein. ^⁎^p < 0.05; ^⁎⁎^p < 0.01; ^⁎⁎⁎^*p* < 0.001. r = 0.47 (*p* < 0.01).

## 4 Discussion

This investigation provides novel insights into the complex relationships between exercise-induced hemodynamic responses, atherogenic lipid profiles, and cardiorespiratory fitness in highly trained endurance athletes. Three key findings emerge: (1) exercise-induced hypertension does not associate with adverse atherogenic lipid subfraction profiles in marathon runners; (2) superior cardiorespiratory fitness demonstrates robust associations with favorable lipid particle characteristics; and (3) fitness-mediated metabolic adaptations appear to supersede exercise-induced blood pressure responses in determining lipid-related cardiovascular risk profiles.

The absence of significant associations between EIH and atherogenic lipid subfractions challenges conventional expectations. While previous studies have demonstrated that EIH can increase the prevalence of coronary artery plaque among middle-aged male marathon runners (Kim et al., 2020), our findings suggest that exercise-induced blood pressure abnormalities may not directly correlate with adverse lipid particle profiles in this population. EIH likely reflects acute hemodynamic stress responses involving transient vascular dysfunction and enhanced sympathetic activation, rather than the chronic metabolic perturbations characteristic of established hypertension (Kim and Park, 2024). The transient nature of exercise-induced blood pressure elevations may be insufficient to induce the metabolic changes that promote atherogenic lipid particle formation. The highly trained nature of our study population may contribute to these findings. Marathon runners with EIH maintain exceptional cardiorespiratory fitness (mean VO₂max: 48.3 mL/kg/min), which may provide metabolic protection against atherogenic lipid profile development despite exercise-induced hemodynamic abnormalities (Leyk et al., 2009).

However, the absence of associations between EIH and atherogenic lipid profiles should not diminish concerns regarding potential cardiovascular risks in athletes with EIH. Recent studies indicate that EIH may contribute to cardiovascular risk via mechanisms independent of lipid metabolism. Gwag et al. (2024) found that excessive blood pressure elevation during exercise is linked with adverse cardiovascular remodeling in marathon runners, including increased left ventricular mass and arterial stiffness, which may develop irrespective of traditional lipid-driven atherogenesis. Furthermore, Burtscher et al. (2022) proposed that repeated cardio-renal stress from extreme endurance exercise could precipitate late cardiovascular sequelae through cumulative hemodynamic load, oxidative stress, and inflammation. These insights suggest EIH is a multifaceted cardiovascular risk factor warranting vigilant clinical attention, beyond lipid-related pathways.

Our findings strongly support the concept that cardiorespiratory fitness represents a dominant determinant of lipid particle quality, independent of exercise-induced blood pressure responses. This aligns with the landmark study by Kawano and colleagues, which demonstrated that improved cardiorespiratory fitness correlates with both quantitative and qualitative changes in small dense LDL particles (Kawano et al., 2009). High CRF promotes favorable lipid particle characteristics through multiple pathways: enhanced lipoprotein lipase activity, improved VLDL clearance, increased LDL particle size, and reduced oxidative stress (Sarzynski et al., 2015; Kraus et al., 2002). These mechanisms likely explain the strong correlations observed between VO₂max and favorable lipid profiles in our study (Wang and Xu, 2017). Halverstadt et al. (2007) supported these findings, showing a similar LDL particle size shift in endurance-trained individuals, further emphasizing CRF's role as a cardiovascular protective factor. Conversely, the HUNT3 Fitness Study reported that individuals with low CRF are more likely to exhibit atherogenic lipid profiles, even when total cholesterol levels are within normal limits (Nodeland et al., 2022). The dramatic difference in atherogenic phenotype prevalence between high-fitness and low-fitness groups (15.4 % vs. 48.0 %, *p* = 0.01) underscores the powerful protective effects of superior cardiorespiratory fitness against lipid-related cardiovascular risk factors.

The interplay between EIH, cardiovascular remodeling, and metabolic health in endurance athletes appears to be more complex than previously recognized. While our data demonstrate that high CRF provides robust protection against atherogenic lipid profiles, this metabolic advantage may not fully mitigate the structural and hemodynamic consequences of repeated exercise-induced blood pressure elevations. Burtscher et al. (2022) proposed that repeated cardio-renal stress from extreme endurance exercise could precipitate late cardiovascular sequelae through cumulative hemodynamic load, oxidative stress, and inflammation, mechanisms that operate independently of lipid metabolism. The bidirectional relationship between kidney function and blood pressure regulation adds another layer of complexity, as chronic vigorous exercise might pose risks for kidney injury or exacerbate existing renal conditions. These considerations suggest that EIH should be viewed as a multifaceted cardiovascular risk factor that requires vigilant clinical attention across multiple physiological domains, not solely through the lens of lipid-related pathways.

These findings have important implications for cardiovascular risk assessment in athletic populations. The results suggest that in highly trained endurance athletes, comprehensive risk stratification should incorporate both lipid-related and non-lipid-related factors. While high CRF provides substantial protection against atherogenic lipid profiles, the presence of EIH warrants additional evaluation for cardiovascular remodeling and renal function. Healthcare providers should implement a multidimensional assessment approach that includes CRF evaluation, exercise blood pressure monitoring, cardiac imaging when indicated, and renal function screening. Exercise prescription strategies should focus on maintaining and improving CRF while monitoring for signs of excessive hemodynamic stress during training.

Several limitations warrant consideration. The cross-*sec*tional design precludes causal inferences. The study was limited to male marathon runners, limiting generalizability to women and other athletic populations. Dietary intake patterns, genetic factors, and inflammatory markers were not assessed, all of which may influence lipid metabolism and cardiovascular remodeling. Cardiac imaging and renal function parameters were not evaluated, precluding direct assessment of cardiovascular and renal sequelae discussed. Future studies should employ prospective longitudinal designs that integrate lipid profiling, cardiovascular imaging, and renal function assessment to elucidate the complex interplay between EIH, CRF, and multisystem cardiovascular risk in diverse athletic populations.

## 5 Conclusions

This study demonstrated that exercise-induced hypertension in marathon runners is not associated with adverse atherogenic lipid subfractions, while superior cardiorespiratory fitness correlates with markedly favorable lipid particle characteristics. These findings suggest that fitness-mediated metabolic adaptations may attenuate lipid-related cardiovascular risks independent of exercise-induced blood pressure responses. However, the absence of lipid-related associations does not preclude other cardiovascular and renal risks associated with EIH, highlighting the multifaceted nature of cardiovascular risk in endurance athletes. The results underscore the importance of comprehensive, multidimensional fitness and cardiovascular assessment in risk stratification for athletic populations and support exercise prescription strategies that optimize cardiorespiratory fitness while monitoring for excessive hemodynamic stress.

## CRediT authorship contribution statement

**Eun Sun Yoon:** Writing – original draft, Visualization, Software, Methodology, Formal analysis. **Jong-Young Lee:** Writing – review & editing, Supervision, Project administration, Investigation, Funding acquisition. **Young-Joo Kim:** Writing – review & editing, Supervision, Project administration, Investigation, Funding acquisition, Data curation, Conceptualization.

## Ethics statement

This study was conducted in accordance with the Declaration of Helsinki and approved by the Institutional Review Board of Sungshin Women's University (SSWUIRB 2023-0921-091). Written informed consent was obtained from all participants.

## Declaration of generative AI and AI-assisted technologies in the writing process

During the preparation of this work, the authors used ChatGPT and Claude to perform grammar checking and language polishing of the manuscript draft. After using these tools, the authors reviewed and edited the content as needed and take full responsibility for the final content of this article.

## Funding

This work was supported by the Ministry of Education of the Republic of Korea and the National Research Foundation of Korea (NRF-2021S1A5A2A03069917).

## Declaration of competing interest

The authors declare that they have no known competing financial interests or personal relationships that could have appeared to influence the work reported in this paper.

## Data Availability

The data supporting the findings of this study are available from the corresponding author upon reasonable request.
